# Home-based online exercise training and chronic, low-grade inflammation in cancer patients after curative surgery: secondary analysis of the randomized controlled multicenter CRBP-TS trial

**DOI:** 10.1007/s00520-025-09608-1

**Published:** 2025-06-11

**Authors:** Johannes Voß, Julian Barisch, René Thieme, Ines Gockel, Uwe Tegtbur, Christian Leps, Martin Busse, Roberto Falz

**Affiliations:** 1 Institute of Sports Medicine and Prevention, Leipzig University, Leipzig, Germany; 2 Department of Visceral, Thoracic and Vascular Surgery, University Hospital Leipzig, Leipzig, Germany; 3 Institute of Sports Medicine, Hannover Medical School, Hannover, Germany

**Keywords:** Curative cancer surgery, Chronic inflammation, Cancer recurrence, Cytokines, Home-based, Exercise

## Abstract

**Purpose:**

Cancer patients who have undergone curative treatment may retain chronic, low-grade inflammation, a condition known to promote carcinogenesis, and, thus, cancer recurrence. We aimed to investigate whether home-based online exercise training can mitigate chronic, low-grade inflammation of cancer patients after curative oncologic surgery.

**Methods:**

We analyzed data sets from 145 patients with breast, prostate, or colorectal cancer after curative surgery in the randomized controlled multicenter CRBP-TS trial. The intervention group was instructed to exercise at least twice weekly via video presentations for 6 months. The control group received no video presentations. We analyzed the modified Glasgow prognostic score (mGPS) and plasma levels of interleukin (IL)-1 beta, IL-2, IL-6, IL-10, IL-12p70, tumor necrosis factor-alpha (TNF-alpha), and interferon-gamma (IFN-gamma) at baseline, after 3 months, and after 6 months. Baseline values were compared to normative values of healthy populations. Mixed-effect models were applied for statistical analysis of intervention effects.

**Results:**

We detected baseline elevations of IL-1 beta, IL-2, IL-6, IL-10, IL-12p70, TNF-alpha, and IFN-gamma compared to normative values of healthy individuals. All patients in each group except for one had an mGPS of 0 at baseline. None of the cytokines revealed any significant interaction effects. After 6 months, all patients had an mGPS of 0.

**Conclusion:**

Cancer patients may exhibit low-grade chronic inflammation after surgery. In this study, home-based online exercise training did not affect low-grade chronic inflammation. Future studies should further investigate the efficacy of home-based online exercise training considering adjunctive therapies, other exercise modalities, and cancer types.

**Trial registration:**

DRKS-ID: DRKS00020499; Registered 17 March 2020.

https://drks.de/search/en/trial/DRKS00020499

**Supplementary Information:**

The online version contains supplementary material available at 10.1007/s00520-025-09608-1.

## Introduction

Cancer is responsible for about every sixth death worldwide [1]. In 2022, female breast cancer had the second highest incidence among cancers with 2.3 million new cases worldwide, followed by colorectal cancer with 1.9 million new cases, and prostate cancer with 1.5 million new cases. Colorectal cancer reveals the second-highest mortality (904,000 estimated deaths). Female breast cancer (666,000 estimated deaths) and prostate cancer (397,000 estimated deaths) place in fifth and eighth position for cancer-related deaths [1]. Tumor resection and perioperative systemic therapies are the primary potential curative treatment strategies. But even if therapy succeeds, systemic post-tumor therapy effects can persist, such as chronic inflammation (CI) [2]. CI might be associated with the recurrence of cancer [3, 4].

The most validated systemic inflammation-based score for the prognosis of cancer is the modified Glasgow Prognostic Score (mGPS) [5]. This score is easy to use, as it only requires C-reactive protein (CRP) and albumin levels [6, 7]. Elevated CRP levels (and, thus, a high mGPS) four or more weeks after surgery reduce recurrence-free and overall survival in colorectal cancer [8], breast cancer [9], and prostate cancer patients [10]. However, CI can also be present without elevated CRP levels. This low-grade CI can be unmasked by analyzing pro- and anti-inflammatory cytokines [11]. Cytokine diagnostics are not part of standard treatment, which makes it difficult to detect low-grade inflammation. Disease recurrence could be prevented more effectively, if the negative side effects of curative treatment, such as low-grade CI, are detected and alleviated early.

Exercise training (ET) is a non-pharmacological option to modulate the inflammatory state and is therefore a potentially promising countermeasure for low-grade CI [12–16]. However, previous meta-analyses have drawn contrary conclusions regarding the impact of ET on the inflammatory state of cancer patients after surgery [17–21]. ET for cancer patients is typically provided at hospitals or rehabilitation centers. Scaling inpatient exercise therapy to large patient groups is currently neither covered nor reimbursed by most healthcare systems, e.g., in Germany, as it is cost-intensive and logistically difficult. This globally reduces the comprehensive and necessary numbers of cancer patients engaging in ET.

The World Health Organization (WHO) recommends that digital health technologies should be added to health systems to improve universal health coverage [22]. A recent review concluded that digital health interventions offer a promising, age-independent approach to improving health outcomes in cancer patients [23]. Providing ways for patients to exercise at home, aided by digital technologies and self-empowerment, has led to physiological and psychological improvements in cancer patients [24–27]. To our knowledge, no study has yet evaluated the effects of home-based online exercise training (HOET) on the inflammatory state of cancer patients after surgery in the short- and mid-term follow-up period.

We aimed to investigate the effects of a 6-month HOET on interleukin (IL)−1 beta (IL-1ß), IL-2, IL-6, IL-10, IL-12p70, tumor necrosis factor-alpha (TNF-α), interferon-gamma (IFN-γ), and the mGPS of colorectal, breast, and prostate cancer patients after surgery. By analyzing these mechanisms, we provide new insights into the unexplored potential of digital technologies for exercise therapy in cancer treatment.

## Materials and methods

### Research design

In this secondary analysis, we relied on data from 145 patients in the randomized controlled multicenter CRBP-TS trial [27]. A comprehensive account of this study design has been published [28]. Ethics approval for the study was obtained from the Ethics Committee of the Medical Faculty, University of Leipzig (reference number 056/20-ek), and from all participating sites.

All participants provided written informed consent. Patients were eligible if they had been diagnosed with colorectal cancer (International Classification of Diseases (ICD) code C18/19/20), breast cancer (ICD code C50), or prostate cancer (ICD code C61), and had undergone curative (R0) surgery at TNM-stages T1 N0M0 to T3 N3M0. We also included colorectal cancer patients at TNM-stage T1 N0M1 in cases of resectable liver metastasis. A postoperative interval from 4 weeks to 6 months following oncologic surgery was analyzed. Exclusion criteria were (1) an ECOG (Eastern Co-operative Oncology Group) score of less than 1, (2) acute cardiac, renal, hepatic, endocrine, bone marrow, or cerebral disorders, and (3) lack of cognitive ability to comprehend and follow the program. After recruitment, all patients were randomly assigned to either the intervention group (IG) or control group (CG) in a 1:1 ratio. Block sizes of two and four were used for stratified randomization by study site and cancer entity. The study interval and data collection period lasted over 6 months. Data were collected at baseline (T1), after 3 months (T2), and after 6 months (T3) following surgery.

### Blood sampling and analysis

We collected ethylenediaminetetraacetic acid blood samples at T1, T2, and T3 to assess IL-1ß, IL-2, IL-6, IL-10, IL-12p70, TNF-α, IFN-γ, CRP, and albumin. After collection, blood samples were centrifuged and then stored at − 80 °C until analysis. We used a custom-made version of the LegendPlex Multi-Analyte Flow Assay Kit (BioLegend, USA) for plasma analysis of IL-1ß, IL-2, IL-6, IL-10, IL-12p70, TNF-α, and IFN-γ according to the manufacturer’s instructions. CRP and albumin were analyzed in the central laboratory of the Institute of Laboratory Medicine, Clinical Chemistry, and Molecular Diagnostics located at the University Hospital Leipzig.

### Modified Glasgow Prognostic Score

We formulated the mGPS with albumin and CRP levels according to established thresholds [5]. A score of 0 was assigned when CRP levels were ≤ 10 mg/L, regardless of the albumin level. A score of 1 was assigned for CRP > 10 mg/L and albumin ≥ 35 g/L, while a score of 2 was assigned for CRP > 10 mg/L and albumin < 35 g/L.

### Digital devices and CRBP-TS application

Participants in the IG and our CG participants were given a tablet (Lenovo Tab M10 TB-X606X; Lenovo, Hongkong, China) with the preinstalled CRBP-TS application to answer questionnaires and to get general information on beneficial lifestyle changes (including diet, exercise, and self-perception). The tablet contained a SIM card (5 GB LTE, Deutsche Telekom AG, Germany) for continuous internet access. All study-related entries on the tablet were saved and transmitted to an electronic case file at the study center. Both groups also received a wearable for activity tracking (Vívoactive 4; Garmin, Olathe, Kansas, USA). Only patients in the IG had access to the exercise videos. They were given an additional chest belt (Polar H7, Polar Electro, Kempele, Finland) to record their heart rate during exercise sessions via Bluetooth low energy. To ensure correct handling of the devices, patient training was carried out by the study staff when the devices were handed out. Technical support during the intervention period was provided for both groups via telephone or remote control as needed.

### Exercise intervention

Patients in the IG were advised to watch and follow at least two exercise videos per week. Each exercise video lasted approximately 30 min and included a general warm-up, an exercise phase, and a cool-down. In the exercise phase, four rounds of five different bodyweight strength-endurance exercises had to be completed (e.g., stepping, squats, push movements, core exercises). Each exercise was performed for 40 s followed by 20 s of recovery. After each completed round, participants rested for 60 s. Exercise videos provided in-depth movement demonstrations with entity-specific cues for proper execution, and options to facilitate or intensify exercises. The video instructor regularly told the participants to choose an intensity level corresponding to a perceived exertion between five and eight on the well-established ten-point Borg Category-Ratio (CR10) scale [29, 30]. After completing an exercise session, the participants rated the session’s perceived exertion (sRPE) on the CR10 scale on the tablet. Exercise videos changed weekly and were individually tailored to each cancer type. They were categorized into three intensity levels corresponding to each patient’s aerobic exercise capacity (obtained from their cardiopulmonary exercise test at baseline). The study site workers adjusted the intensity categories if the participant’s aerobic exercise capacity increased significantly at T2. Intensity categories were also adjusted if the sRPE was consistently reported below five or above eight. In the event of unscheduled exercise breaks, patients were sent reminders through short message services to maintain adherence.

### Statistical analysis

We analyzed the sampling distribution with the Shapiro–Wilk test. Inferential statistics were conducted with an intention-to-treat approach, including all randomized participants with an existing baseline measurement. Values below the detection limit were replaced by values corresponding to half of the detection limit. This imputation method is easy to use and not significantly inferior to more complex imputation methods [31, 32]. Data from participants with missing data were included under the missing-at-random assumption. We applied a mixed-effects model with a repeated measurement structure (estimated using restricted maximum likelihood). Values at baseline and after 6 months were the dependent variables. All values were log(10)-transformed to reflect the skewness of the data. Moreover, all analyses were also done using the original values and presented in supplementary Table 1. The categorical time covariate and the randomization arm were determined as fixed effects. Interactions were analyzed for group and time (categorical). We set an intercept for subjects as a random effect. Within the mixed-effects model, we calculated 95% confidence intervals (CI) and *p*-values to compare groups. We conducted a sensitivity analysis for time differences within groups (using a paired t-test for dependent samples) of patients with complete paired baseline and 6-month follow-ups. All analyses were two-sided, and the significance level was set at *p* = 0.05. All values are expressed as medians and percentiles unless otherwise stated. For mGPS, we counted the number of respective values at the start of the study and after 6 months for both groups. We analyzed the data with IBM SPSS Statistics (Version 29; IBM, Armonk, NY, USA) and displayed them with GraphPad Prism (Version 9; GraphPad Software Inc., CA, USA).

## Results

Our results reflecting changes in exercise capacity, body composition, quality of life, program adherence, and lifestyle have been reported previously [27, 33].

For our analysis, we had to exclude three of the 148 participants in the CRBP-TS trial because of lacking blood sample data at all visits. At T3, data were available for 62 participants in the IG and 59 participants in the CG. Table 1 illustrates patient demographics and clinical data at baseline. Participants in the IG engaged with 2.1 (SD = 1.1) exercise videos per week. Both groups had similar activity levels [27].

**Table 1:** Baseline characteristics of the study population

Variables	Intervention group (*n* = 75)	Control group (*n* = 70)
Age (years)	55 ± 10	55 ± 12
Sex, no		
Female	44 (59%)	42 (60%)
Male	31 (41%)	28 (40%)
Height (cm)	172 ± 8	171 ± 8
Body composition		
Weight (kg)	79.5 ± 14.8	75.1 ± 14.2
Fat mass (kg)	24.6 ± 10.3	20.8 ± 7.5
Lean body mass (kg)	55.2 ± 11.2	53.3 ± 10.3
Body mass index (kg/m^2^)	26.6 ± 4.7	25.1 ± 4.1
Waist-to-hip ratio	0.88 ± 0.11	0.87 ± 0.10
Cancer entity, no		
Colorectal cancer	10 (13%)	8 (11%)
Breast cancer	42 (56%)	41 (59%)
Prostate cancer	23 (31%)	21 (30%)
Comorbidities, no		
Diabetes mellitus type 2	4	2
Arterial hypertension	14	16
Obesity (BMI > 30 kg/m^2^)	16	10
Cardiovascular diseases	2	3
Hypothyroidism	7	8
Asthma	2	2
Arthritis	9	3
Cancer medication, no		
Estrogen receptor modulator	9	14
Monoclonal antibody	2	1
Aromatase inhibitors	8	5
Chemotherapy medication	3	2

Values are presented as means and standard deviations. *SAE* serious adverse event, *BMI* body mass index

### Inflammatory biomarkers

At baseline, we observed elevated IL-1β (Mdn: 32.2 pg/ml, IQR: 18.0–62.8), IL-2 (Mdn: 1.54 pg/ml, IQR: 0.59–1.56), IL-6 (Mdn: 14.5 pg/ml, IQR: 10.6–30.0), IL-10 (Mdn: 6.44 pg/ml, IQR: 2.86–13.31), IL-12p70 (Mdn: 6.59 pg/ml, IQR: 2.19–22.0), TNF-α (Mdn: 25.1 pg/ml, IQR: 9.3–43.4), and IFN-γ (Mdn: 7.20 pg/ml, IQR: 2.75–19.9) in both groups compared to healthy individuals [33]. C-reactive protein and albumin were unremarkable in both groups. Normative values are presented in Table 2 [34–36]. Our mixed-effects analysis revealed no significant interaction effects for the HOET between groups and times. Time effects were evident for IL-1β (*p* = 0.001), IL-10 (*p* = 0.005), IL-12p70 (*p* = 0.001), TNF-α (*p* = 0.001), and IFN-γ (*p* = 0.04). We found no group effects. Our sensitivity analysis revealed significant increases in IL-1β (*p* = 0.03) and IL-12p70 (*p* = 0.003) in the CG, and in IL-12p70 (*p* = 0.003) in the IG. Table 2 displays the values of the inflammation markers at baseline and after 6 months in the IG and CG.

**Table 2:** Inflammation marker levels at baseline and after 6 months

	Median (25 th/75 th percentile) [sample size]								Time effect^a^	Group effect^a^	Interaction effect^a^
**Parameter** (normative value)	**Intervention group** **____________________**			**Control group** **_____________________**				Diff^b^ 6 month IG vs. CG (95% CI)	*p*	*p*	Group × Time *p*
	**pre**	**6 mo**	**MD**^**c**^	**pre**	**6 mo**	**MD**^**c**^					
**IL-1β,** pg/ml (n. d)	43.4 (18.0/83.4) [75]	43.4 (22.8/94.6) [62]	5.0 (− 12.0/30.2) [62]	27.4 (18.0/43.4) [70]	36.9 (18.0/64.7) [59]	3.3* (− 9.7/33.5) [59]	− 5.6 (− 24 to 13)		0.001*	0.10	0.60
**IL-2,** pg/ml (0.21)	1.5 (0.6/2.2) [75]	1.1 (0.6/2.9) [62]	0.0 (− 1.0/1.0) [62]	1.5 (0.6/1.5) [70]	1.1 (0.6/2.0) [59]	0.0 (− 0.7/0.2) [59]	1.1 (− 2.0 to 4.2)		0.89	0.45	0.58
**IL-6,** pg/ml (n. d.)	14.5 (10.6/31.5) [75]	16.7 (10.6/46.6) [62]	0.0 (− 4.1/13.4) [62]	14.5 (10.6/24.5) [70]	16.7 (10.6/25.4) [59]	0.0 (− 6.3/16.2) [59]	1.1 (− 17 to 20)		0.06	0.41	0.70
**IL-10,** pg/ml (0.54)	7.1 (4.2/16.6) [75]	7.3 (4.2/19.0) [62]	0.9 (− 4.9/8.5) [62]	6.2 (2.7/12.1) [70]	6.4 (5.1/15.5) [59]	0.9 (− 1.4/7.2) [59]	1.1 (− 6.3 to 8.6)		0.005*	0.34	0.76
**IL-12p70,** pg/ml (n. d.)	6.6 (2.2/23.0) [75]	7.2 (5.0/23.0) [62]	1.4* (0.0/9.2) [62]	6.4 (2.2/12.8) [70]	7.2 (3.6/23.0) [59]	0.7* (0.0/16.8) [59]	2.1 (− 11 to 15)		0.001*	0.36	0.48
**TNF-α,** pg/ml (0.27)	31.2 (9.3/58.4) [75]	31.2 (23.1/98.6) [62]	5.0 (− 18.3/30.2) [62]	24.8 (9.3/31.2) [70]	25.1 (12.9/46.6) [59]	0.0 (14.8/24.0) [59]	6.5 (− 16 to 29)		0.001*	0.11	0.51
**IFN-γ,** pg/ml (4.27)	8.2 (2.6/25.2) [75]	9.0 (5.0/30.2) [62]	0.4 (− 6.4/14.5) [62]	7.2 (2.8/13.8) [70]	7.2 (5.0/15.9) [59]	1.5 (− 5.0/7.5) [59]	− 2.7 (− 19 to 14)		0.04*	0.13	0.80
**CRP** mg/L (< 3.0)	1.5 (0.7/2.5) [75]	0.9 (0.6/2.1) [62]	− 0.1* (− 1.0/0.1) [62]	1.0 (0.6/1.9) [70]	0.9 (0.6/1.6) [59]	0.0 (− 0.3/0.3) [59]	0.4 (− 3.4 to 4.2)		0.26	0.19	0.40
**Albumin** g/L (43.0)	45.1 (43.4/46.9) [75]	45.1 (42.9/46.1) [62]	− 0.2 (− 2.0/1.3) [62]	45.3 (43.5/47.3) [70]	44.5 (43.5/46.3) [59]	− 0.5 (− 2.5/1.7) [59]	0.2 (− 0.7 to 1.1)		0.31	0.44	0.94

*pre* baseline, *mo* month, *MD* median difference

*Significant difference (*p* < 0.05)

^a^Main effects of mixed-effects model with log(10)-transformed values

^b^Estimates of differences between group changes over time

^c^Sensitivity analysis: results of the complete case analysis considering all available data

**Table 3:** mGPS scores in IG and CG at baseline and after 6 months

Score	Intervention group		Control group	
	**pre, no**	**6 mo, no**	**pre, no**	**6 mo, no**
0	74 (98.7%)	62 (100%)	69 (98.6%)	59 (100%)
1	1 (1.3%)	0 (0%)	1 (1.4%)	0 (0%)
2	0 (0%)	0 (0%)	0 (0%)	0 (0%)

Note: *mGPS* modified Glasgow prognostic score, *pre* baseline, *mo* month.

Score 0: CRP ≤ 10 mg/L regardless of albumin; Score 1: CRP > 10 mg/L and albumin ≥ 35 g/L; Score 2: CRP > 10 mg/L and albumin < 35 g/L.

**Fig. 1:**
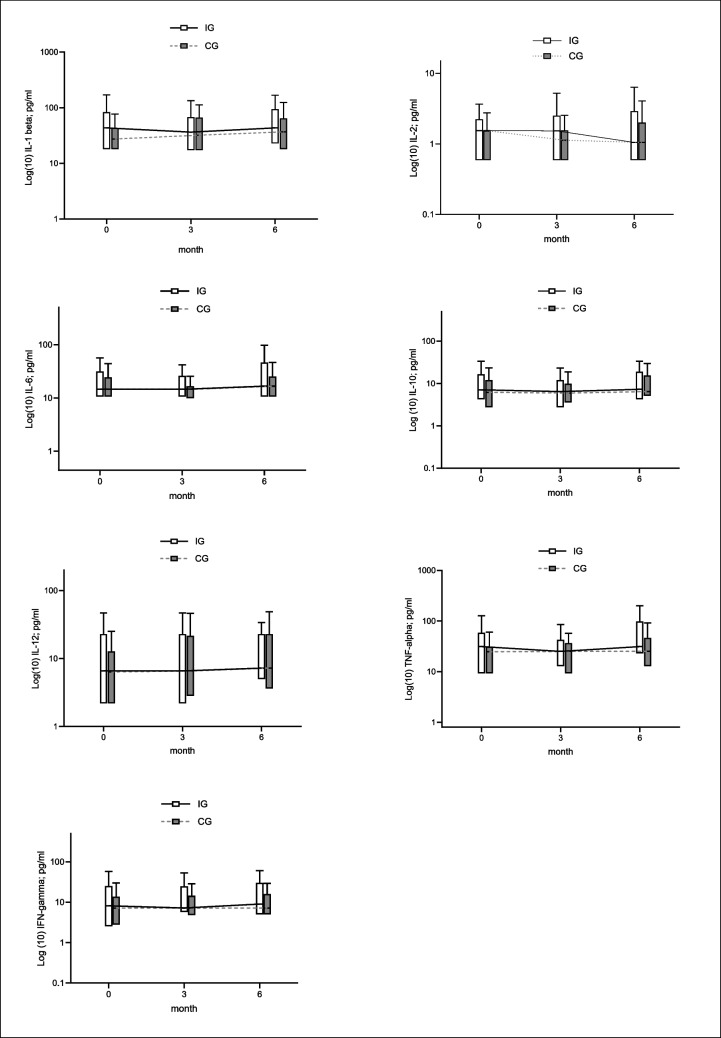
Development of IL-1β, IL-2, IL-6, IL-10, IL-12, TNF-α, and IFN-γ levels in IG and CG during the intervention

Note: Y-axis logarithmized (log10); Box [IQR with median-line]; Whiskers [1.5 × IQR]; *IG* intervention group, *CG* control group.

Figure 1 shows the development of IL-1β, IL-2, IL-6, IL-10, IL-12p70, TNF-α, and IFN-γ during the intervention period.

### Modified Glasgow Prognostic Score

We calculated the modified Glasgow prognostic score (mGPS) since it is the most validated systemic inflammation-based prognostic score for cancer patients [5]. Table 3 shows the results of the mGPS in IG and CG at T1 and T3.

## Discussion

This is the first analysis evaluating the effects of a 6-month HOET on the inflammatory state of cancer patients after curative surgery. In this secondary analysis of our randomized controlled CRBP-TS trial, we evaluated the effects of HOET on the cytokines IL-1ß, IL-2, IL-6, IL-10, IL-12p70, TNF-α, IFN-γ, and the mGPS of 145 cancer patients. Our results on established parameters of postoperative systemic inflammation and its influence by physical exercise underline the notion that the inflammatory state of cancer patients after surgery should be closely monitored. The findings support HOET as an important, safe, and non-pharmacological treatment option.

Our study has yielded two main results:

Baseline inflammatory state.

First, we identified an mGPS of 0 in 98.7% of the IG participants and 98.6% of the CG participants. At first glance, this result indicates an apparent absence of chronic inflammation. However, cytokine analysis revealed the elevated proinflammatory cytokines IL-1ß, IL-6, IL-12p70, TNF-α, and IFN-γ as well as slightly elevated anti-inflammatory cytokines IL-2 and IL-10 in both groups compared to a healthy population [37–39]. There has been contradictory research evidence regarding whether cancer patients suffer from CI after surgery [39]. Our findings add to the evidence that cancer patients retain low-grade CI even after successful R0 resection. Furthermore, CRP and the mGPS were not elevated in almost all subjects, supporting the notion that the inflammatory state of cancer patients should be monitored more closely. Otherwise, normal CRP values or an mGPS of 0 could conceal chronic, low-grade inflammation. Ultimately, we acknowledge that an increase in cytokines would also be conceivable in the case of a chronic infection during a long-term (complicated, e.g., chronic wound infection) postoperative course, although this has not been systematically recorded.

Effects of HOET on the inflammatory state.

Second, we observed that a 6-month HOET failed to affect the IL-1ß, IL-2, IL-6, IL-10, IL-12p70, TNF-α, and IFN-γ levels in our participants. Khosravi et al. meta-analyzed 26 exercise-oncologic studies that primarily included breast, prostate, and colorectal cancer survivors [19]. They reported that combined aerobic and resistance training triggers minor reductions in pro-inflammatory markers (SMD − 0.3), with CRP (SMD − 0.5) and TNF-α (SMD − 0.3) being most sensitive to change. However, although we could not reproduce their findings, several causes should be considered.

Subjects in our IG completed the exercise program an average of 2.1 times per week, while the average training volume in the meta-analyzed studies by Khosravi et al. was 3.5 training sessions per week [19]. The exercise frequency in our study therefore may have been too low to improve our participants’ inflammatory state.

Note that only three studies in the meta-analysis of Khosravi et al. applied home-based exercise training [40–42]. In those studies, no digital technologies were applied, and only isolated cytokines, such as IL-6 or TNF-α were examined in a single type of cancer. Comparability with our study is therefore limited.

Chronic inflammation is a complex interplay of several cytokines and their downstream products. An extensive inflammatory marker profile should thus be obtained rather than individual parameters. So far, the effects of HOET on an extensive inflammatory marker profile have only been described by Moon et al. [42]. In their pilot study with 15 prostate cancer patients, they evaluated the effects of a 24-week HOET on IL-1α, IL-5, IL-6, IL-12, TNF-α, and IFN-γ. They observed no significant differences in these cytokines from baseline to the study’s conclusion, neither in the intervention group (*n* = 9) nor in the control group (*n* = 6). We examined a similar marker profile in our study. Our analysis revealed similar results and relied on a larger sample than the study of Moon et al. Unlike in their trial, we also included different cancer entities, limiting the comparability of our results with theirs [42]. We instead provide new knowledge on the dynamics of cytokines during HOET in cancer patients after curative surgery.

### Limitations

Our findings should be interpreted carefully due to the following limitations. First, the inclusion times after surgery varied among the patients, due to patients’ convalescence and time of recovery as a prerequisite for onboarding. All patients, however, were covered in the period lasting from 4 weeks to 6 months after surgery, which is the most relevant period for chronic inflammation in postoperative cancer patients [8]. Second, systemic therapy, which varies among patients, can obviously affect inflammation and might have influenced our results accordingly [43]. We were unable to retrospectively differentiate between the influences of systemic therapy. Third, we grouped colorectal, breast, and prostate cancer patients as a general cohort of cancer patients. The side effects of cancer treatment can vary depending on the cancer type. Not considering subgroup analyses for different cancer types could have masked the impact of HOET specific to those cancer types. Yet the subgroups’ sample sizes would have been too small for a robust statistical analysis. Fourth, we replaced values below the detection limit with their detection limit divided by 2. More complex imputation procedures could have hindered a potential distortion of the results caused by the imputation. However, we would have expected only a slight distortion from our method compared to more complex methods [44].

## Conclusions

In summary, we provide new evidence on the effects of HOET on the inflammatory state in a colorectal, breast, and prostate cancer cohort after curative surgery. Our findings indicate that cancer patients may exhibit low-grade chronic inflammation after surgery. Home-based online exercise training following cancer surgery did not influence low-grade chronic inflammation. It remains unclear whether home-based online exercise training can generally influence low-grade chronic inflammation. Future studies should further investigate the efficacy of home-based online exercise training considering adjunctive therapies, other exercise modalities, and cancer types.

## Supplementary Information

Below is the link to the electronic supplementary material.

Supplementary file1 (DOCX 18.6 KB)

## Data Availability

The datasets analyzed during the present study can be obtained from the corresponding author on reasonable request.
