# Supplementary material for: Home-based online exercise training and chronic, low-grade inflammation in cancer patients after curative surgery: secondary analysis of the randomized controlled multicenter CRBP-TS trial
Source: Support Care Cancer. 2025 Jun 11;33(7):566. doi: 10.1007/s00520-025-09608-1 (PMC12159120; doi:10.1007/s00520-025-09608-1)
Supplement: Supplementary file 1 — Supplementary file1 (DOCX 18.6 KB) [file 520_2025_9608_MOESM1_ESM.docx]

**Appendix**

|  | Median (25^th^ / 75th percentile) [sample size] | | |  | | | | |  | Time effect^a^ | | | Group effect^a^ | | Inter-action effect^a^ | |
| --- | --- | --- | --- | --- | --- | --- | --- | --- | --- | --- | --- | --- | --- | --- | --- | --- |
| **Parameter**  (normative value) | **Intervention group**  **____________________** | | | | **Control group**    **_____________________** | | | | Diff^b^ 6 month IG vs. CG (95% CI) | | p | | p | | Group x Time  p | |
|  | **pre** | **6 mo** | **MD** | | **pre** | **6 mo** | **MD** |  | | |  |  | |  | |  |
| **IL-1ß,**  pg/ml  (n. d) | 43.4 (18.0/ 83.4) [75] | 43.4 (22.8/ 94.6) [62] | 5.0  (-12.0/ 30.2)  [62] | | 27.4 (18.0/ 43.4) [70] | 36.9 (18.0/ 64.7) [59] | **3.3***  (-9.7/ 33.5)  [59] | -5.6 (-24 to 13) | | | **0.03** | | **0.02** | | 0.37 | |
| **IL-2,**  pg/ml  (0.21) | 1.5  (0.6/ 2.2)  [75] | 1.1  (0.6/ 2.9)  [62] | 0.0  (-1.0/ 1.0)  [62] | | 1.5  (0.6/ 1.5)  [70] | 1.1  (0.6/ 2.0)  [59] | 0.0  (-0.7/ 0.2)  [59] | 1.1 (-2.0 to 4.2) | | | 0.37 | | 0.83 | | 0.29 | |
| **IL-6,**  pg/ml  (n. d.) | 14.5 (10.6/ 31.5) [75] | 16.7 (10.6/ 46.6) [62] | 0.0  (-4.1/ 13.4)  [62] | | 14.5 (10.6/ 24.5) [70] | 16.7 (10.6/ 25.4) [59] | 0.0  (-6.3/ 16.2)  [59] | 1.1 (-17 to 20) | | | 0.36 | | 0.16 | | 0.78 | |
| **IL-10,**  pg/ml  (0.54) | 7.1  (4.2/ 16.6) [75] | 7.3  (4.2/ 19.0) [62] | 0.9  (-4.9/ 8.5)  [62] | | 6.2  (2.7/ 12.1) [70] | 6.4  (5.1/ 15.5) [59] | 0.9  (-1.4/ 7.2)  [59] | 1.1 (-6.3 to 8.6) | | | 0.22 | | 0.09 | | 0.76 | |
| **IL-12p70,** pg/ml  (n. d.) | 6.6  (2.2/ 23.0) [75] | 7.2  (5.0/ 23.0) [62] | **1.4***  (0.0/ 9.2)  [62] | | 6.4  (2.2/ 12.8) [70] | 7.2  (3.6/ 23.0) [59] | **0.7***  (0.0/ 16.8)  [59] | 2.1 (-11 to 15) | | | 0.08 | | 0.23 | | 0.49 | |
| **TNF-α,**  pg/ml  (0.27) | 31.2 (9.3/ 58.4) [75] | 31.2 (23.1/ 98.6) [62] | 5.0  (-18.3/ 30.2)  [62] | | 24.8 (9.3/ 31.2) [70] | 25.1 (12.9/ 46.6) [59] | 0.0  (14.8/ 24.0)  [59] | 6.5 (-16 to 29) | | | 0.09 | | **0.04** | | 0.55 | |
| **IFN-γ,**  pg/ml  (4.27) | 8.2  (2.6/ 25.2) [75] | 9.0  (5.0/ 30.2) [62] | 0.4  (-6.4/ 14.5)  [62] | | 7.2  (2.8/ 13.8) [70] | 7.2  (5.0/ 15.9) [59] | 1.5  (-5.0/ 7.5)  [59] | -2.7 (-19 to 14) | | | 0.18 | | **0.03** | | 0.95 | |
| **CRP**  mg/L  (< 3.0) | 1.5  (0.7/ 2.5)  [75] | 0.9  (0.6/ 2.1)  [62] | **-0.1***  (-1.0/ 0.1)  [62] | | 1.0  (0.6/ 1.9)  [70] | 0.9  (0.6/ 1.6)  [59] | 0.0  (-0.3/ 0.3)  [59] | 0.4 (-3.4 to 4.2) | | | 0.22 | | 0.85 | | 0.66 | |
| **Albumin**  g/L  (43.0) | 45.1 (43.4/ 46.9)  [75] | 45.1 (42.9/ 46.1)  [62] | -0.2  (-2.0/ 1.3)  [62] | | 45.3 (43.5/ 47.3)  [70] | 44.5 (43.5/ 46.3)  [59] | -0.5  (-2.5/ 1.7)  [59] | 0.2 (-0.7 to 1.1) | | | 0.28 | | 0.47 | | 0.92 | |

**Table 1**: Inflammation marker levels at baseline and after six months without log(10)-transformation

Note: *pre* baseline, *mo* month, *MD* median difference

*Significant difference (p < 0.05)

^a^ Main effects of mixed-effect model

^b^Estimates of differences between group changes over time

^c^Sensitivity analysis: results of the complete case analysis considering all available data
